# Supplementary material for: The Predictive Value of miR-16, -29a and -134 for Early Identification of Gestational Diabetes: A Nested Analysis of the DALI Cohort
Source: Cells. 2021 Jan 15;10(1):170. doi: 10.3390/cells10010170 (PMC7830355; doi:10.3390/cells10010170)
Supplement: Supplementary file 1 [file cells-10-00170-s001.pdf]

cells MDPI

Supplementary figures and tables

Supplementary Table 1: Small RNAs and their association with GDM and primers used in the study

Name	Link to GDM and/or purpose	Forward 5′	Reverse 5′	Ref.
**c.el-miR-39**	Spike-in at cDNA synthesis	ACACTCCAGCTGGGTC ACCGGGTGTAAATC	CTCAACTGGTGTCGTG GAGTCGGCAATTCAGT TGAGCAAGCTGA	
**U6**	Endogenous reference gene	CTCGCTTCGGCAGCAC A	AACGCTTCACGAATTT GCGT	
**Universal Reverse primer (UPR)**	Applied in most assays		TGGTGTCGTGGAGTCG	
**at-miR-159a**	Spike-in at RNA isolation	ACACTCCAGCTGGG TTTGGATTGAAGGG	CTCAACTGGTGTCGTG GAGTCGGCAATTCAGT TGAGTAGAGCTCC	
**miR-223-3p**	Up-regulated in GDM	ACACTCCAGCTGGGTG TCAGTTTGTCAAAT	CTCAACTGGTGTCGTG GAGTCGGCAATTCAGT TGAGTGGGGTAT	[35,38]
**miR-16-5p**	Up-regulated in GDM	ACACTCCAGCTGGG TAGCAGCACGTAAAT	CTCAACTGGTGTCGTG GAGTCGGCAATTCAGT TGAG CGCCAATA	[34,55, 57]
**miR-122-5p**	Down-regulated in GDM Dysregulated in metabolic syndrome	ACACTCCAGCTGGG TGGAGTGTGACAATG	CTCAACTGGTGTCGTG GAGTCGGCAATTCAGT TGAG CAAACACC	[36]
**miR-29a-3p**	Dysregulated in GDM	ACACTCCAGCTGGGTA GCACCATCTGAAAT	CTCAACTGGTGTCGTG GAGTCGGCAATTCAGT TGAGTAACCGAT	[33,34, 38]
**miR-433-3p**	Located in DLK1-DIO3 locus	ACACTCCAGCTGGG ATCATGATGGGCTCC	CTCAACTGGTGTCGTG GAGTCGGCAATTCAGT TGAG ACACCGAG	
**miR-134-5p**	Located in DLK1-DIO3 locus AND Up-regulated in GDM	ACACTCCAGCTGGG TGTGACTGGTTGACC	CTCAACTGGTGTCGTG GAGTCGGCAATTCAGT TGAG CCCCTCTG	[27]
**miR-330-3p**	Up-regulated in GDM	ACACTCCAGCTGGG GCAAAGCACACGGCC T	CTCAACTGGTGTCGTG GAGTCGGCAATTCAGT TGAG TCTCTGCA	[34,39]
**miR-103-3p**	Dysregulated in pregnancy	ACACTCCAGCTGGG AGCAGCATTGTACAGG	CTCAACTGGTGTCGTG GAGTCGGCAATTCAGT TGAG TCATAGCC	[36]

Supplementary Figure 1: Study flow chart

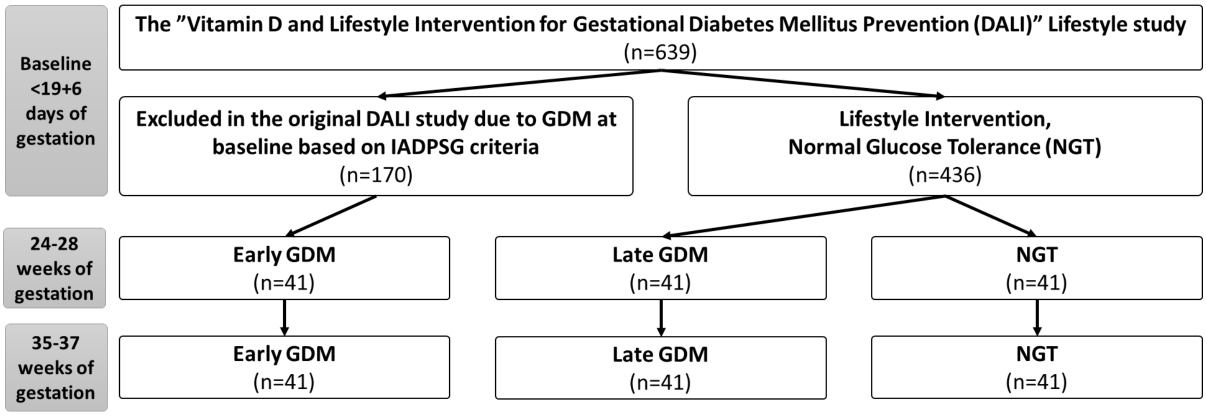

Supplementary Figure 1: Study flow chart. A total of 123 women were included in the current study. Gestational diabetes mellitus (GDM) was classified according to the time of GDM diagnosis; early GDM (before 20 weeks of gestation) and late GDM (at 24-28 weeks of gestation), while controls were normal glucose tolerance (NGT) throughout pregnancy. GDM diagnosis at 35-37 weeks were not used in the current study.

Supplementary Figure 2: **Circulating small RNAs in GDM cases and controls.**

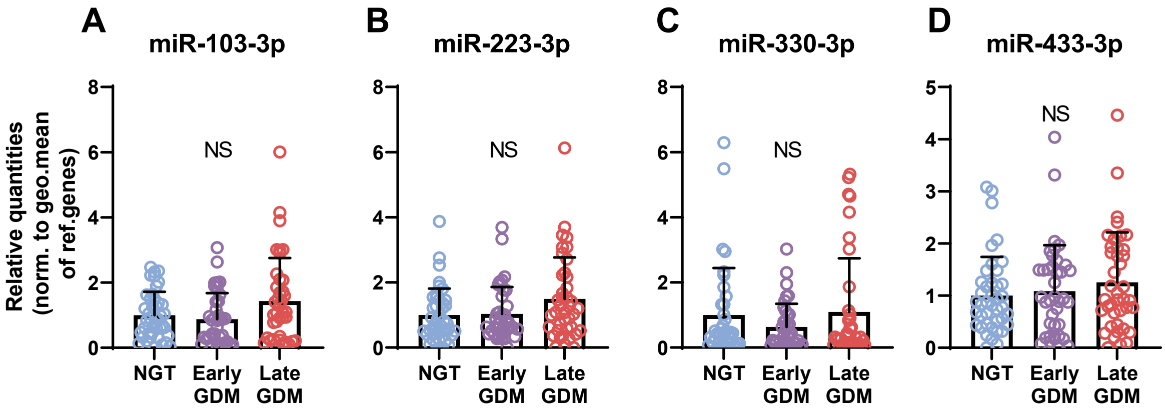

Supplementary Figure 2: Relative mean quantities with standard deviations of serum microRNAs expressed in the three groups (A-D). Data were normalized against the geometric mean of snRNA U6, ath-miR-159 and c.el.miR-39. All indicated p values were determined by independent t-test on logarithmical transformed data with Bonferroni correction for multiple testing.

Supplementary Table 2: Receiver Operate Curve characteristics

Non-coding RNA	Area under the curve (AUC)	Comparison	p-value
**miR-16, -29a and -134**	0.717	NGT vs Late GDM	**0.001**
**Fasting glucose**	0.687	NGT vs Late GDM	**0.004**
**1h glucose**	0.563	NGT vs Late GDM	0.334
**2h glucose**	0.681	NGT vs Late GDM	**0.005**
**HbA1c**	0.597	NGT vs Late GDM	0.160
**miR-29a-3p**	0.698	NGT vs Late GDM	**0.002**
**miR-16-5p**	0.687	NGT vs Late GDM	**0.005**
**miR-134-5p**	0.654	NGT vs Late GDM	**0.017**
**miR-122-5p**	0.602	NGT vs Late GDM	0.116
**miR-29a-3p**	0.577	NGT vs Early GDM	0.239
**miR-16-5p**	0.464	NGT vs Early GDM	0.582
**miR-134-5p**	0.543	NGT vs Early GDM	0.510
**miR-122-5p**	0.428	NGT vs Early GDM	0.273
**miR-29a-3p**	0.597	Late GDM vs Early GDM	0.136
**miR-16-5p**	0.679	Late GDM vs Early GDM	**0.007**
**miR-134-5p**	0.555	Late GDM vs Early GDM	0.399
**miR-122-5p**	0.647	Late GDM vs Early GDM	**0.028**

Supplementary Table 2: ROC curve analysis for distinguishing GDM cases (either early or late GDM) from healthy pregnant normal glucose tolerant (NGT) controls. ROC, receiver operating characteristic curve.

Supplementary Figure 3: Receiver Operating Characteristic Curves in GDM cases and healthy pregnant controls.

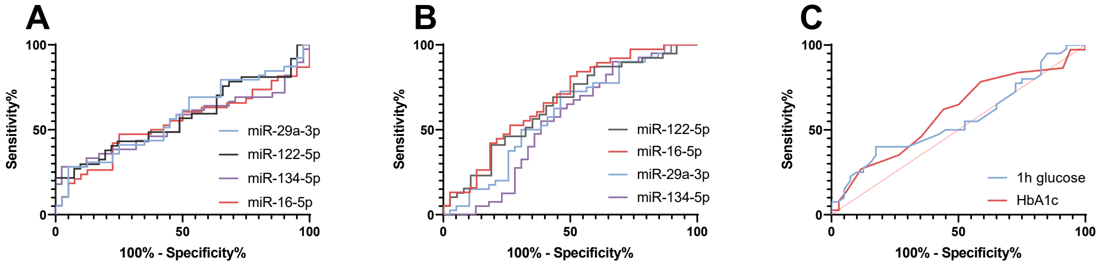

Supplementary Figure 3: Receiver operator characteristic curves based on microRNA. Shown are four microRNAs A) ROC curve for single miRNAs comparing women with normal glucose tolerance and their capability of diagnosis GDM before 20 weeks of gestation. None of the miRNAs were significant. B) ROC curve analysis between pregnant women excluded at baseline due to GDM and pregnant women who develop GDM later. The AUC for miR-16-5p (0.679, p=0.007) and miR-122-5p (0.647, p=0.028) could distinguish between the two GDM cases. C) ROC curve analysis of 1-hour glucose (AUC=0.563, p=0.334) and HbA1c (AUC=0.599, p=0.150) for distinguishing between NGT and late GDM.

Supplementary Table 3: Predicted enriched genes and pathways linked to GDM and miR-16-5p, -29a-3p, 122-5p and 134-5p.

Gene ID	Pathway	miR-16-5p	miR-29a-3p	miR-122-5p	miR-134-5p
**MAT2A**	S-adenosylmethionine biosynthesis		x		x
**G6PC**	Phenylethylamine degradation		x	x	
**CMPK1**	De novo pyrimidine ribonucleotides biosythesis	x	x		
**CPS1**	De novo pyrimidine ribonucleotides biosythesis		x		x
**IGF1**	Insulin/IGF pathway-protein kinase B signaling cascade	x	x		
**FOXO3**	Insulin/IGF pathway-protein kinase B signaling cascade, PI3 kinase pathway		x	x	
**PIK3CA**	Insulin/IGF pathway-protein kinase B signaling cascade, PI3 kinase pathway, Ras Pathway, VEGF signaling pathway, Angiogenesis, T cell activation, FGF signaling pathway		x		x
**FBXW7**	Notch signaling pathway	x	x		
**NCOR2**	Notch signaling pathway	x	x		
**NOTCH2**	Notch signaling pathway, Angiogenesis	x	x		
**KRAS**	PI3 kinase pathway, Ras Pathway, VEGF signaling pathway, Angiogenesis	x			x
**PAK3**	Ras Pathway, Angiogenesis, T cell activation			x	x
**MAP2K6**	Ras Pathway, p38 MAPK pathway, FGF signaling pathway	x	x		
**VEGFA**	VEGF signaling pathway, Angiogenesis	x	x		
**FRS2**	Angiogenesis, FGF signaling pathway	x	x		
**CALM3**	T cell activation		x	x	
**CREB1**	p38 MAPK pathway			x	x

Supplementary Table 3. Predicted enriched genes and pathways linked to GDM. TargetScan (v.7.2, human) was used together with Panther Classification System (v.15.0).

Supplementary Figure 4: **Association of miR-16-5p and miR-29a-3p with fetal macrosomia**

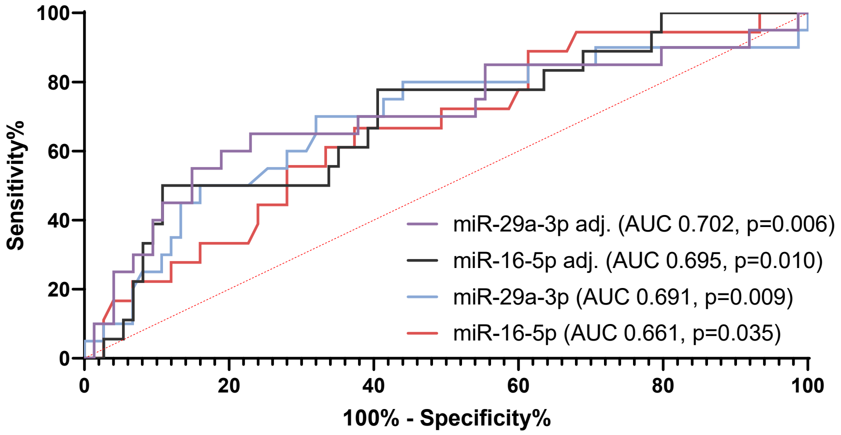

Supplementary Figure 4: ROC curve analysis between pregnant women who delivered a macrosomic baby (>4000g) and pregnant women who did not. All women included, N=95-93. AUCs were adjusted (adj.) for maternal age and BMI and offspring sex.

Supplementary Figure 5: **Sex differences between the selected miRNAs.**

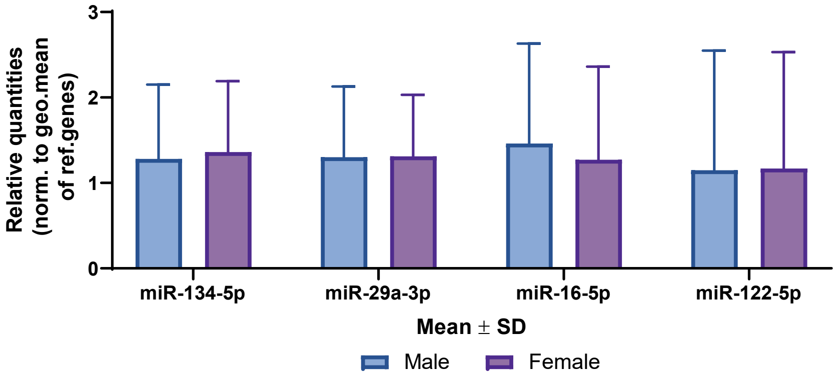

Supplementary Figure 5: Relative mean quantities with standard deviations of serum microRNAs expressed in women with either male or female offspring. Data were normalized against the geometric mean of snRNA U6, ath-miR-159 and c.el.miR-39. All indicated p values were determined by independent t-test on logarithmical transformed data with Bonferroni correction for multiple testing.
